# Interplay between Selenium, Selenoproteins and HIV-1 Replication in Human CD4 T-Lymphocytes

**DOI:** 10.3390/ijms23031394

**Published:** 2022-01-26

**Authors:** Olivia M. Guillin, Caroline Vindry, Théophile Ohlmann, Laurent Chavatte

**Affiliations:** 1 Centre International de Recherche en Infectiologie (CIRI), 69007 Lyon, France; olivia.guillin@ens-lyon.fr (O.M.G.); caroline.vindry@ens-lyon.fr (C.V.); 2 Institut National de la Santé et de la Recherche Médicale (INSERM) Unité U1111, 69007 Lyon, France; 3 Ecole Normale Supérieure de Lyon (ENS), 69007 Lyon, France; 4 Université Claude Bernard Lyon 1 (UCBL1), 69622 Lyon, France; 5 Centre National de la Recherche Scientifique (CNRS), Unité Mixte de Recherche 5308 (UMR5308), 69007 Lyon, France

**Keywords:** selenoproteome, HIV-1, viral infection, glutathione peroxidase, thioredoxin reductase, SELENOS, SELENOO, primary T cells, Jurkat, SupT1, translational control

## Abstract

The infection of CD4 T-lymphocytes with human immunodeficiency virus (HIV), the etiological agent of acquired immunodeficiency syndrome (AIDS), disrupts cellular homeostasis, increases oxidative stress and interferes with micronutrient metabolism. Viral replication simultaneously increases the demand for micronutrients and causes their loss, as for selenium (Se). In HIV-infected patients, selenium deficiency was associated with a lower CD4 T-cell count and a shorter life expectancy. Selenium has an important role in antioxidant defense, redox signaling and redox homeostasis, and most of these biological activities are mediated by its incorporation in an essential family of redox enzymes, namely the selenoproteins. Here, we have investigated how selenium and selenoproteins interplay with HIV infection in different cellular models of human CD4 T lymphocytes derived from established cell lines (Jurkat and SupT1) and isolated primary CD4 T cells. First, we characterized the expression of the selenoproteome in various human T-cell models and found it tightly regulated by the selenium level of the culture media, which was in agreement with reports from non-immune cells. Then, we showed that selenium had no significant effect on HIV-1 protein production nor on infectivity, but slightly reduced the percentage of infected cells in a Jurkat cell line and isolated primary CD4 T cells. Finally, in response to HIV-1 infection, the selenoproteome was slightly altered.

## 1. Introduction

The human immunodeficiency virus (HIV) is an enveloped, linear, positive-sense single-stranded RNA virus that belongs to the family of *Retroviridae* (Group VI), genus *Lentivirus*. HIV is the etiological agent of acquired immunodeficiency syndrome (AIDS) and is responsible for a weakened immune system, as it infects immune cells [1]. There are two types of HIV (HIV-1 and HIV-2) that differ in their epidemiological and pathological properties [1]. In contrast to HIV-2 that is mostly confined to West Africa, HIV-1 has spread worldwide, due to its improved infectivity and virulence [2]. There are currently more than 37 million people infected with human immunodeficiency virus (HIV), causing about 1.5 million deaths every year (http://www.who.int/hiv/en/, accessed on 1 December 2021). HIV infection is now considered a chronic disease that requires intensive treatment and can present a variable clinical course. HIV-1 infects immune cells that harbor the CD4 receptor and a co-receptor belonging to the chemokine receptor family (CCR5 and CXCR4) [1]. Therefore, cells infected by HIV-1 are CD4 T lymphocytes, monocytes, macrophages and dendritic cells. A decline in CD4 T cells is characteristic of the progression of HIV infection and is often used as a prognostic marker. No vaccine is currently available, but an effective combination of drugs, called antiretroviral therapy (ART), has been developed to lower the viral load and increase the CD4 T-cell count [3,4]. Worldwide, about a quarter of HIV-infected people are still not receiving ART. In addition, the emergence of drug resistant viruses remains a threat to the effectiveness of ART. The replication but also the latency of the virus is extremely variable from one cell type to another. Lentiviruses are characterized by a long incubation period after the primo infection, which is highly variable from one patient to another. During this time, humans infected with HIV are under chronic oxidative stress and present nutritional deficiencies, particularly with regard to selenium [5]. In HIV-infected people, lower selenium levels have been associated with lower CD4 T-cell counts, faster progression of AIDS, and a 20% increase in the risk of death [6,7]. The beneficial effects of selenium nutritional supplementation were reported in a few studies by a decrease in viral burden, an increase in the time of progression to AIDS and an indirect increase in the number of CD4 T cells [8,9,10]. These findings remained significant even after correction for the ART regimen [10]. Interestingly, a long-time treatment with ART (more than 2 years) improved selenium levels as compared with HIV-infected patients with a shorter time of exposure to ART or those who are not receiving any treatment [11]. Since the discovery of HIV in the 1980s, several established T-cell lines (including Jurkat, SupT1 and CEM) were found permissive to this virus and were instrumental for investigating the molecular mechanisms of viral replication and virus–host interactions that occur in the course of infection. In contrast to these established cultured cell lines, resting primary CD4 T cells isolated from donors represent a more physiological model relevant to HIV infection, but these cells are more difficult to grow, infect, and they must be activated to increase their susceptibility to HIV infection. Even when stimulated, the infection efficiency of primary cells rarely reaches the levels observed in established T-cell lines. In the present study, we used both cellular models: cultured T cells, SupT1 and Jurkat, and primary CD4 T cells that were isolated from different healthy donors.

Selenium is an essential trace element implicated in many facets of human health and disease. This element is often evoked in the context of immune function and infectious diseases caused by viruses or bacteria [5,12]. The current example of the COVID-19 pandemic, due to the SARS-CoV-2 viral infection, confirms this finding with an association between low levels of selenium in body fluids and poor prognosis of infected patients hospitalized in intensive care units, as reviewed in [13]. Several studies showed that nutritionally deficient humans or animals were more susceptible to a wide variety of infections [5]. The mechanism is probably more complex than expected in the sense that nutritional deficiency does not impact only the host immune response, but also the viral pathogen itself. For instance, dietary selenium deficiency that induces oxidative stress in the host can alter the genome of a virus so that a normally benign or mildly pathogenic virus becomes highly virulent. This phenomenon has been particularly well described in animal models for influenza and coxsackie viruses [14,15,16,17], but has not been investigated for other viral models yet. Consequently, it was hypothesized that selenium deficiency could participate in the genetic evolution of a wide variety of viruses and their pathogenicity by a mechanism that remains to be elucidated.

Most of selenium’s beneficial effects are attributed to its presence as selenocysteine, a rare amino acid, in a small, but vital group of redox enzymes that constitute the selenoproteins. They are implicated in antioxidant defense, redox homeostasis, redox signaling and possibly other cellular processes [18,19,20]. Among the selenoproteins, glutathione peroxidases (GPXs) and thioredoxin reductases (TXNRDs) are well characterized [21,22]. While the GPXs are part of the antioxidant defenses that reduce a wide variety of peroxides using glutathione (GSH) as a cofactor [23], the TXNRDs are NADPH-dependent reductases that control the redox balance of a broad range of substrates, including proteins (thioredoxins or glutaredoxin 2) but also small selenium- and sulfur-containing molecules (lipoic acid, DTNB, selenite, selenocystine, etc.) [24]. In addition, a third of the selenoproteome is localized at the endoplasmic reticulum (ER), where they participate in protein folding, calcium homeostasis and ER-stress response [25,26,27,28,29]. These ER-resident selenoproteins include SELENOF, SELENOI, SELENOK, SELENOM, SELENON, SELENOS, SELENOT and DIO2.

Therefore, as a family of redox enzymes, selenoproteins are thought to play important functions in the immune system, as reviewed in [30], although experimental data are still awaited. The selenoproteome is encoded by 25 genes and is primarily controlled by selenium bioavailability, which induces prioritization of selenoprotein biosynthesis [18,31,32]. The hierarchical regulation of the selenoproteome by other exogenous stimuli, cellular sensors or pathophysiological conditions is poorly understood [33,34,35,36,37,38]. This hierarchy of response induced by variations of the selenium concentration is very specific to each tissue or cell line and relies primarily on a translational control strategy. Indeed, the insertion of selenocysteine in selenoproteins is based on a non-conventional translational mechanism that is unique in many aspects. Indeed, selenocysteine is encoded by UGA, which is normally a stop codon. As the first addition to the genetic code in 1991, selenocysteine is, therefore, often referred to as the 21st amino acid [39,40]. Thus, the cell has developed a singular strategy to recode UGA as a selenocysteine in selenoprotein mRNAs, while it continues to be used as a stop codon for all other cellular mRNAs. This efficiency of UGA recoding as selenocysteine is rather low (between 1% and 5%), even under optimal conditions of selenium levels, and most often results in a truncated protein, with the UGA codon being read as a stop codon [31]. In theory, this poorly efficient UGA codon can be seen as a premature stop codon by the dedicated nonsense mediated decay (NMD) surveillance machinery that safeguards the quality of transcripts in eukaryotic cells [41]. However, even though several selenoprotein mRNAs follow the prerequisites of NMD rules, only a few mRNA transcripts are targeted by this mechanism, and this only occurs upon selenium deprivation and in specific cell lines [34,42]. Additionally, recent experiments using ribosome profiling, a method that allows precise location of the ribosome position on mRNAs, showed that UGA-selenocysteine recoding event is a limiting step, which is very sensitive to selenium level variation [43,44,45].

Although low serum selenium levels are associated with HIV progression in many epidemiological studies, cellular and molecular evidence is still lacking to clarify the role of selenium and selenoproteins in viral infection. Moreover, very little information is available on the expression and regulation of the selenoproteome in CD4 T cells, either originating from lymphoma or from healthy donors. To date, the only relevant study was performed in vitro and reported a modification of the pattern of selenoprotein expression in response to HIV infection in lymphocytes, as revealed by ^75^Se radioactive isotope labeling [46]. Based on the migration on SDS-PAGE and radioactive signal intensity, it was suggested that TXNRD1, GPX4 and GPX1 were downregulated in Jurkat cells after HIV-1 infection in favor of one or more low-molecular-weight selenocompounds. However, these experiments were done at a time when only a few selenoproteins were characterized. As the mammalian selenoproteome is now complete [21,47,48] and the physiology of selenium is better understood, this prompted us to investigate further the role of selenium and selenoproteins during HIV infection at the molecular level. Here, we performed a detailed analysis of selenoproteome expression at the mRNA and protein levels in response to varying selenium concentrations both in primary and cultured T cells. We also investigated the replication and pathogenicity of HIV-1 produced in control or selenium-supplemented media. Finally, we studied how the selenoproteins expression in CD4 T cells isolated from healthy donors were affected by HIV infection.

## 2. Results

### 2.1. Comparison of Selenoprotein mRNA Expression Pattern in Established and Primary CD4 T-Cells

**Figure 1:**
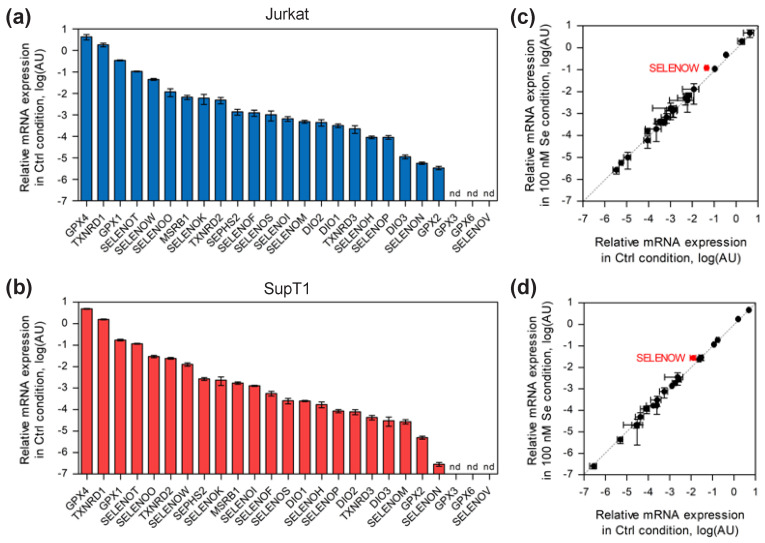
Analysis of selenoprotein mRNA levels in Jurkat and SupT1 in control conditions and in response to 100 nM selenium supplementation of culture media (three days). The geometrical mean of four housekeeping genes (HPCB, RPS13, HRPT, and GAPDH) was used to normalize mRNA abundance. In all panels, mRNA levels are represented in logarithmic scales. The values are given in Table S1. Selenoprotein mRNA levels in control medium (Ctrl) are represented for Jurkat (**a**) and SupT1 (**b**), from most to least abundant (from left to right). To evaluate the impact of selenium supplementation on steady state levels of selenoprotein mRNAs, the values obtained in selenium supplemented conditions (100 nM) were plotted as a function of values obtained with unsupplemented ones (Ctrl) for Jurkat (**c**) SupT1 (**d**) cells (±standard deviation). The experiments were done in biological triplicate and in technical triplicate. The selenoprotein genes with significant changes are labeled in red and the statistical analyses are given in Table S1.

**Figure 2:**
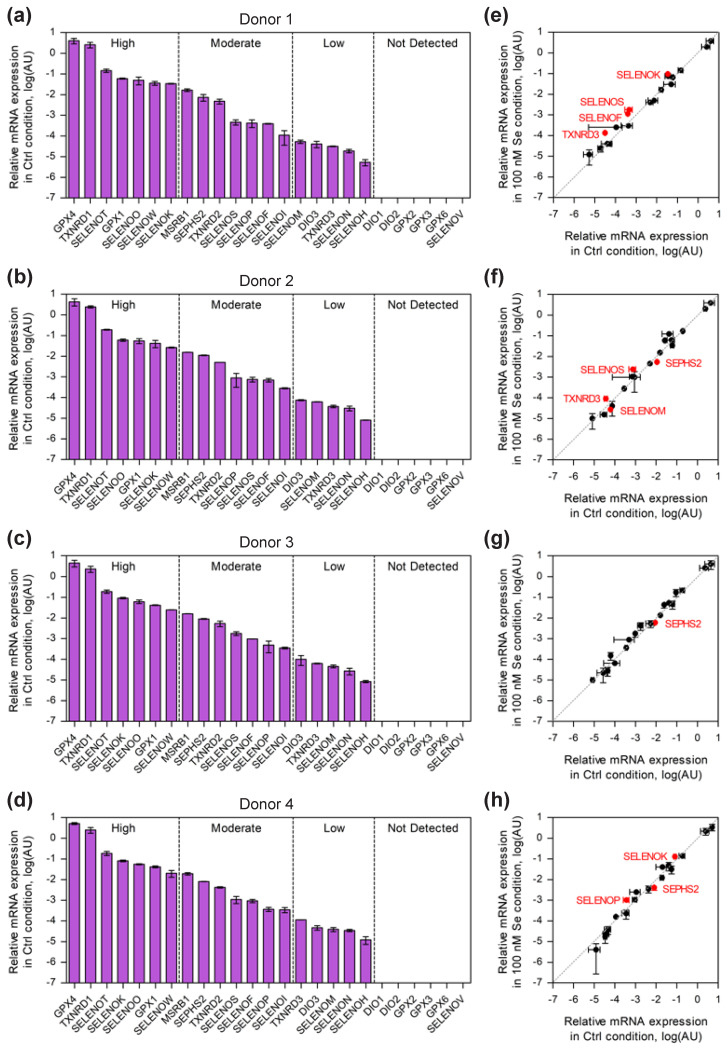
Analysis of selenoprotein mRNA levels in CD4-T cells isolated from donors in control conditions (Ctrl) and selenium-supplemented (100 nM) conditions after three days. The levels are represented in logarithmic scales. The transcripts from four different donors were measured and normalized similarly to what has been done for Jurkat and SupT1 extracts. The mRNAs expressed in control conditions were represented as histograms (**a**–**d**) and arbitrarily separated in high, medium and low abundance by dashed lines. Additionally, the values obtained in selenium-supplemented conditions (100 nM) were plotted as a function of values obtained with unsupplemented ones for every donor (±standard deviation) (**e**–**h**). The values are given in Table S2. The experiments were done in biological duplicates and in technical triplicates. The selenoprotein genes with significant changes are labeled in red and the statistical analyses are given in Table S2.

**Figure 3:**
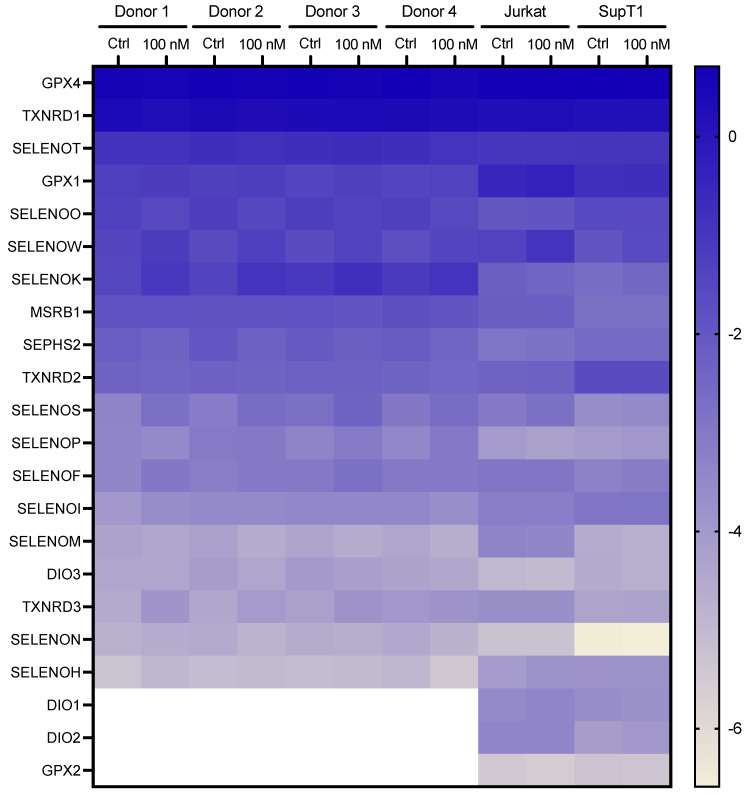
Heatmap representation of selenoprotein mRNA levels (in logarithmic scales) as a function of T-cell types (donors 1 to 4, Jurkat and SupT1) and growth conditions (Ctrl or supplemented with 100 nM of selenium). The values are given in Tables S1 and S2.

Among the selenoprotein family, in mammals, several members, such as GPX1, GPX4 and TXNRD1, are ubiquitously and abundantly expressed, while others are highly tissue specific, such as GPX2, GPX3, GPX6, SELENOV and, to a lesser extent, deiodinases. Although it is now well established that the selenoproteome is highly regulated by selenium availability in several cellular models, very little is known about the expression pattern of these selenoproteins and their response to selenium supplementation in human immune cells, and particularly T lymphocytes. Therefore, as a prerequisite, we used RT-qPCR to analyze the levels of selenoprotein mRNAs in established human cell lines (Jurkat and SupT1) and in primary CD4 T cells purified from four different donors as shown in Figure 1, Figure 2 and Figure 3, respectively. The cells were grown in culture medium supplemented or not with 100 nM of sodium selenite, and total RNA was extracted after three days. Cellular extracts were then referred to as 100 nM and Ctrl, respectively. Of note, the Ctrl media contained an endogenous concentration of 11 nM of selenium, which was essentially provided by the fetal calf serum; thus, after supplementation, the final concentration was 111 nM of total selenium. Interestingly, this selenium concentration of the Ctrl media is rather low and is often considered selenium deficient [49]. In our experiments, the majority of the 25 selenoprotein genes were detected in the various T-lymphocyte extracts. Three classes of mRNAs (SELENOV, GPX3 and GPX6) were below the threshold of detection in all cell types, and three more transcripts (DIO1, DIO2 and GPX2) could not be detected in primary CD4 T cells.

Figure 1 and Figure 2 show the distribution pattern of the selenoproteins in Ctrl conditions for the two cell lines and primary cells used in this study. Interestingly, a comparison of the data obtained in the two lymphoma-derived T cells (Figure 1) shows numerous similarities. Firstly, the four most abundant selenoprotein mRNAs present in Jurkat and SupT1 were identical: GPX4, TXNRD1, GPX1 and SELENOT. While the first three proteins are often expressed at high level in most tissues, this is not the case for SELENOT, which is generally restricted to endocrine organs and embryogenesis. Overall, the expression of selenoprotein mRNAs was comparable between these two cellular lymphoma-derived T-cell lines, with two notable exceptions for the TXNRD2 mRNA, which was five times more expressed in SupT1 than in Jurkat cells and for SELENOM mRNA, which was, conversely, 18 times less expressed in SupT1 than in Jurkat (Figure 1a,b and Table S1). We then extended our analysis to primary CD4 T cells isolated from four different healthy donors and activated with magnetic beads coupled to anti-CD3 and anti-CD28 antibodies. As illustrated in Figure 2 and Table S2, a very similar expression pattern of selenoprotein gene expression was observed in the Ctrl conditions, in the four different extracts, although with subtle variations depending on the donor. To simplify the comparison between the four different donors, we arbitrarily separated the transcripts in high, moderate and low abundance categories (see Figure 2a–d). For these three classes of transcripts, we observed variation between the four donor samples. As shown in Figure 2, the most abundant transcripts, which included GPX4, TXNRD1, SELENOT, GPX1, SELENOO, SELENOW, and SELENOK, were similar in all four donors, but with significant donor-specific variation in their rankings, particularly for GPX1 and SELENOK mRNAs (Figure 2a–d). This contrasts with the expression of mRNA transcripts within the moderate and low abundance categories that were very similar between the four different donors. Finally, when comparing these donor-derived CD4 T-cells with lymphoma-derived cell lines, common features emerged, most notably the high expression of GPX4, TXNRD1 and SELENOT transcripts. The high expression of SELENOK mRNA appeared to be more specific to primary cells, although its ranking was variable among donors.

Then, we analyzed the effect of selenium addition on the expression of selenoprotein transcripts. Indeed, the addition of selenium in the growth media is a well-characterized enhancer of selenoprotein expression in many cell lines, although with, generally, a limited impact at the transcriptional level [31,42]. As illustrated in Figure 1c,d and Figure 2e–h, a limited number of selenoprotein transcripts were sensitive to selenium supplementation. As previously observed in non-immune cell lines, only SELENOW transcript was found to be sensitive to selenium supplementation in T cells, although this can be considered statistically significant (*p* < 0.05) only in Jurkat and SupT1 (see Tables S1 and S2 for the quantitative values). Moreover, it appeared that selenium-specific regulation of transcripts was more pronounced in primary CD4 T cells than in lymphoma-derived cell lines, although with a subtle donor-specific pattern. Thus, among the selenoprotein genes that were selectively altered in response to selenium variation in one or several primary cells, we found SELENOK, SEPHS2, SELENOS, SELENOF, SELENOM, SELENOP and TXNRD3.

A heatmap representation of selenoprotein mRNAs levels obtained in various cell types and selenium levels is shown in Figure 3. Although our data show a significant degree of homogeneity in the expression of the mRNAs in the primary CD4 T cells from different donors, it also reveals significant differences in the transcriptional pattern between primary CD4 T cells and established cell lines. This is not surprising, as Jurkat and SupT1 are derived from lymphoma, and it has been shown that selenium metabolism could be altered in cancer cells [18]. In addition, the level of several selenoproteins, including TXNRD1 and GPX2 [50,51], have been linked to different cancers. It was, therefore, not surprising to observe a different pattern of selenoprotein mRNA expression in cancer cells compared to primary cells.

### 2.2. Selenium-Dependent Hierarchy of Selenoprotein Expression Levels in Established and Primary CD4 T-Cells

**Figure 4:**
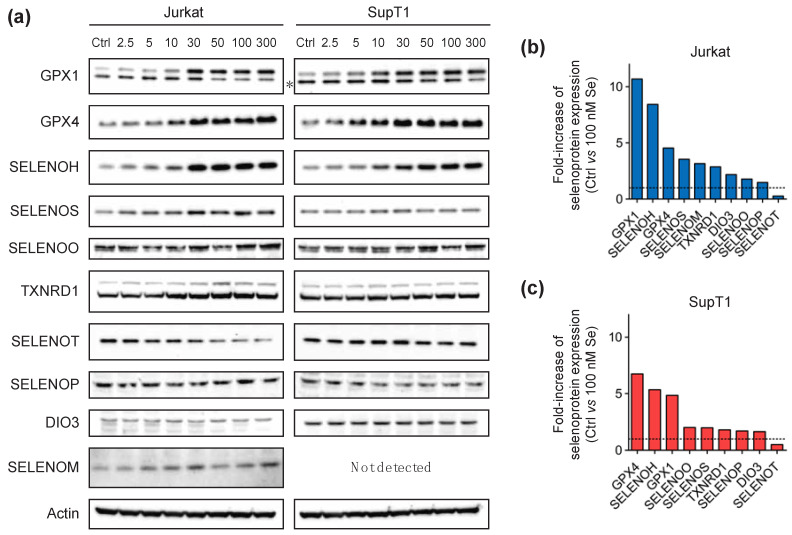
Analysis of selenoprotein expression in Jurkat and SupT1 cells in response to different selenium supplementations of the culture media (in nM) after three days. The results from a representative experiment are shown in (**a**). The values are given in Table S4. The fold-increase in expression of each selenoprotein in response to selenium supplementation was calculated between the value at 100 nM over that at Ctrl. These ratios were plotted from the highest to the lowest for Jurkat (**b**) and SupT1 (**c**) cells. The dotted line represents a fold-increase value of one.

**Figure 5:**
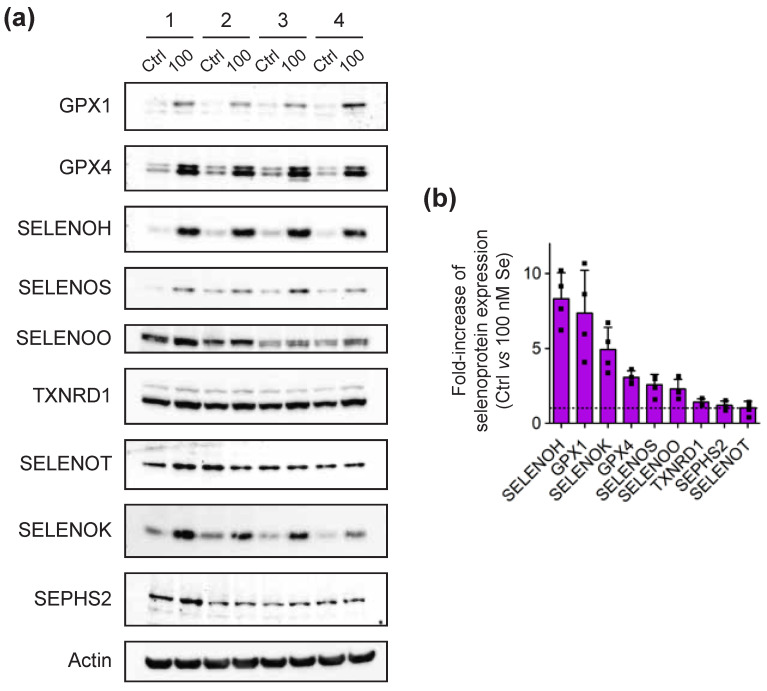
Analysis of selenoprotein expression in CD4 T cells isolated from four different donors in control (Ctrl) and selenium-supplemented (100 nM) conditions over three days. The results from a representative experiment are shown in (**a**). The values are given in Table S4. (**b**) The fold-increase in expression of each selenoprotein in response to selenium supplementation was calculated between the value at 100 nM over that at Ctrl. The average value (±standard deviation) of the four donors was represented by a bar, but the individual value of each donor was indicated by a square. The dotted line represents a fold-increase value of one.

**Figure 6:**
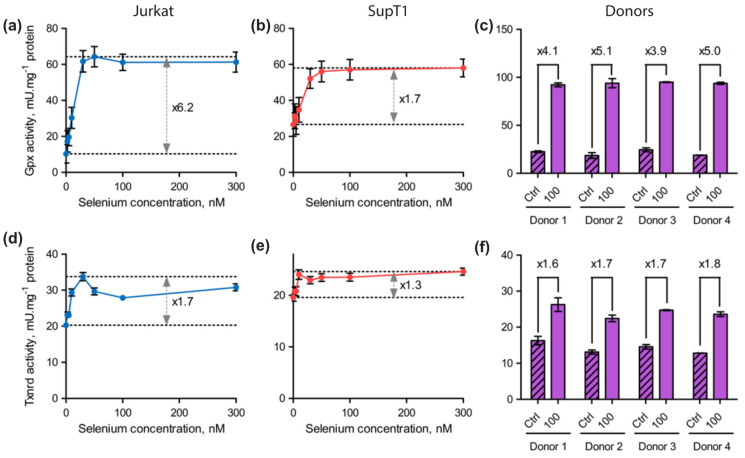
Evaluation of GPX (**a**–**c**) and TXNRD (**d**–**f**) enzymatic activities in protein extracts from various T cell types (Jurkat, SupT1 and the four donors) cultured with different concentrations of supplemented selenium. The GPX activities were represented for Jurkat (**a**) (*n* = 1), SupT1 (**b**) (*n* = 1) and the four donors (**c**) (*n* = 2) for the different growth conditions. The TXNRD activities were represented for Jurkat (**d**) (*n* = 1), SupT1 (**e**) (*n* = 1) and the four donors (**f**) (*n* = 2) for the different growth conditions. The experiments were done in technical triplicates. The differences between the lowest and highest values were indicated by an arrow or a bar (±standard deviation), with the corresponding fold-change factor beside or above.

Our data show, so far, that the level of expression of selenoprotein mRNAs was poorly affected by selenium variations in the culture medium in human T lymphocytes. Thus, the next step was to measure protein expression, as it is expected that selenoprotein production could be more sensitive to selenium addition as previously observed in non-immune cell lines [33,34,42]. Indeed, as described in these previous reports, most of this regulation occurs at the level of translation. This was confirmed by ribosome profiling in animal models fed diets containing different amounts of selenium, where it was observed that the UGA-selenocysteine recoding event was affected by the level of selenium [44,45]. Well-described selenium responders were GPX members and several ER-located selenoproteins [33,34,42]. These proteins are considered ‘stress-response’ selenoproteins, in contrast to the ones that are less sensitive to selenium variations, which are referred to as ‘housekeeping’ members. These less sensitive selenoproteins often include the family of TXNRDs. As was done previously, the different established human cell lines (Jurkat and SupT1) and primary CD4 T cells purified from four different donors were grown in culture medium supplemented, or not, with sodium selenite. For the Jurkat and SupT1 cell lines, we used eight different growth media with increasing selenium concentration up to 300 nM. Due to the finite number of cell divisions of donor-derived cells, and the amount of protein extracts necessary for biochemical studies, only two conditions could be imposed for primary cells, Ctrl and 100 nM, respectively. For all these conditions, cell extracts were harvested after three days of growth in these respective media. Cell extracts were analyzed by Western blots (Figure 4 and Figure 5), but also by enzymatic assays that specifically measure glutathione peroxidase and thioredoxin reductase activities (Figure 6).

Immunodetection of proteins by Western blot was not as easy as the detection of transcripts by RT-qPCR, as it was highly dependent on the availability and quality of antibodies raised against human proteins. Most selenoproteins are rather small in size, which reduces the probability of having immunogenic regions. We tried to detect 19 selenoproteins for which commercially antibodies were available (see Table S3), with several of them previously validated in other cell lines [33,34,37,38,52]. Among them, 10 selenoproteins were unambiguously detected in, at least, one of the T-cell derived extracts. As illustrated in Figure 4 and Figure 5, seven selenoproteins, that included TXNRD1, GPX1, GPX4, SELENOH, SELENOO, SELENOS and SELENOT, were detected in both lymphoma-derived and primary CD4 T cells. The expression of others was more cell-line specific. SELENOM was only detected in Jurkat (Figure 4a), while SELENOK and SEPHS2 were only detected in primary cells (Figure 5a). Several of them were highly sensitive to selenium supplementation of the culture medium. As illustrated in Figure 4b,c and Figure 5b, selenium was able to stimulate the expression of most of them with the notable exception of SELENOT, whose expression decreased with selenium supplementation in both Jurkat and SupT1 cell extracts. However, in extracts derived from primary cells, SELENOT had a variable response to selenium supplementation. In any case, our data further confirm That the control growth conditions that contain 10% FCS and are typically used in most laboratories, is limiting for the expression of several selenoproteins.

As observed in many cell lines, the GPX members expressed in T cells, namely GPX1 and GPX4, were among the most responsive to changes in selenium concentration, confirming their place in the family of ‘stress-related’ selenoproteins. Reactivity to selenium supplementation was further confirmed by the results of the GPX activity assays that are presented in Figure 6. Indeed, we noted a significant increase in GPX activities in response to selenium supplementation in all cell types. In Jurkat and SupT1, the activities reached a plateau from 30 nM of selenium being added, and it remained high up to the maximal concentration used (300 nM); see Figure 6a,b. As such, a 100 nM selenium concentration was expected to reach the highest GPX activity in primary cells. When comparing the GPX activities in control and supplemented primary cell extracts, we observed a 4- to 5-fold increase between Ctrl and 100 nM conditions (Figure 6c). This stimulation of GPX activity was similar between the four different donors and correlates with the level of GPX1 and GPX4 overexpression observed by Western blots shown in Figure 5b. Concerning TXNRD1, which is considered a ‘housekeeping’ member, it was found to be weakly but significantly stimulated by selenium supplementation in all cell types tested here (Figure 4, Figure 5 and Figure 6). These data are in good agreement with previously published reports done in several other non-immune cell lines [33,34,42]. Our data in CD4 T cells were further confirmed by enzymatic assays that are shown in Figure 6c,f. In conclusion, the expression of the selenoproteome is tightly regulated by the selenium level of the culture media in immune cells at a translational level.

### 2.3. Selenium Levels Did Not Affect HIV-1 Replication in Jurkat Cells but Modified the Proportion of Infected Cells

**Figure 7:**
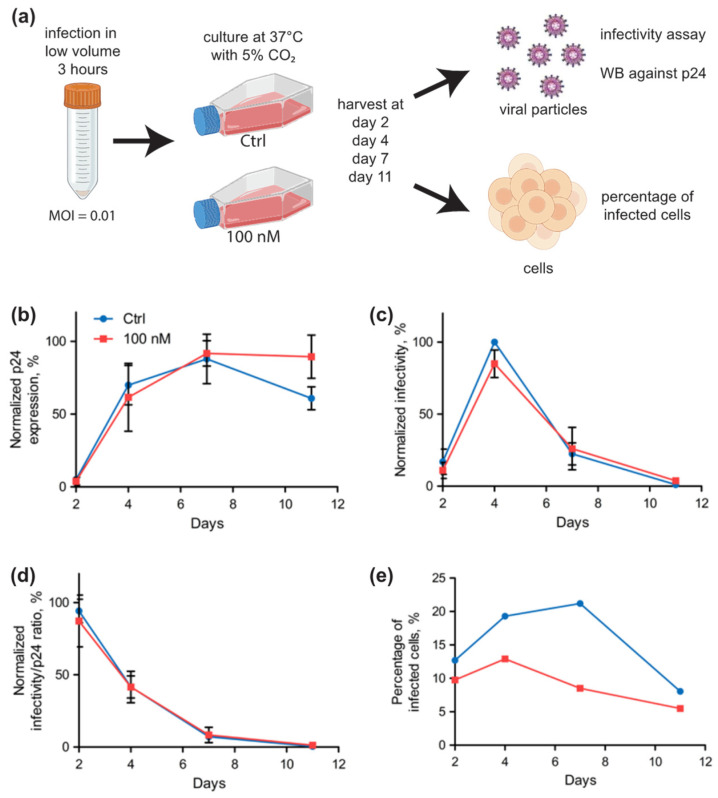
Kinetics of HIV-1 infection of Jurkat cells in control (Ctrl) or 100 nM supplemented conditions. (**a**) Schematic of the experimental procedure used to follow the different parameters of viral production and cell infection during eleven days. The culture media were collected at different time points and analyzed for the levels of HIV-1 p24 capsid protein by Western blot (WB) (**b**) and infectivity of TZM-bl cells (**c**). The anti-p24 Western blots were assessed with biological duplicates, and levels of p24 were expressed relative to the maximum value, arbitrarily set at 100%. The infectivity assays were assessed with biological duplicates in technical triplicates. The maximum value was arbitrarily set as 100%. (**d**) The ratio between infectivity and p24 values were calculated and plotted as a function of time, the maximum value was arbitrarily set as 100%. (**e**) The percentage of infected cell was evaluated in one experiment by immuno-labeling of fixed cells using anti-HIV-1 p24 antibodies coupled with FITC followed by flow cytometry analysis.

We then investigated whether selenium had an effect on virus replication and whether the viral particles produced were equally infectious. We started with Jurkat cells, which have been widely used to study the mechanisms of HIV replication and which seemed to be slightly more sensitive to selenium than SupT1 cells in terms of selenoprotein expression. Please note that selenium was supplemented three hours post-infection. Jurkat cells were infected with the fully replicative HIV-1-NL4.3 at a low moiety of infection (MOI = 0.01) in order to follow the production of HIV viral particles over a period of 11 days (Figure 7a). The time required for HIV to complete replication and to produce a new generation of virus is 24 h [53]. Thus, if we follow the replication kinetics over several replication cycles (about 10 generations in 11 days), a cumulative effect could emerge. After infection of the Jurkat cells, samples were harvested at different times and analyzed for the presence of virus particles and their infectivity. In parallel, the progression of viral infection was evaluated by monitoring synthesis of the HIV-1 capsid protein, also known as p24, by immunodetection on Western blot. HIV-1 p24 is the result of proteolytic cleavage of the polyprotein precursor p55 Gag (Pr55Gag), which occurs after the release of virus particles from the infected cell during the maturation step. Although the quantification of p24 is a good indicator of the amounts of HIV-1 particles released in the media from infected cells, this does not reflect the infectivity of the viruses produced, which is dependent from many other viral parameters. Thus, it was critical to also measure the infectious nature of the produced particles since it is well established that many defective HIV particles can be produced and released in vitro. The infectivity of the produced viral particles was evaluated by testing their ability to infect TZM-bl reporter cells, a HeLa-derived cell line that expresses the CD4 receptor, CXCR4 co-receptor and a luciferase gene, whose expression is driven by a LTR HIV-1 promotor. Therefore, by measuring p24 amounts and ability to infect TZM-bl reporter cells, we could measure both viral production and infectivity under different cellular growth conditions. In parallel, the proportion of infected cells within the total cell population was evaluated by detecting p24 positive cells by flow cytometry after immunological labeling.

As shown in Figure 7b, we found that p24 levels increased during the first few days, post-infection, in a similar manner with or without the addition of selenium to the culture media. The peak of p24 levels was attained at day 7, and this was followed by a significant drop in viral protein amount in control conditions. Interestingly, the infectivity of the particles released in the culture media was virtually identical throughout the kinetics for both cellular growth conditions, with a maximum at day 4 followed by a decrease to reach an almost complete decrease at day 11 (Figure 7c). These data suggest that selenium does not have a significant effect on HIV-1 particle production, neither in terms of quantity nor infectivity as revealed by the ratio representing the infectivity over the level of p24, both detected in the media (Figure 7d). The percentage of infected cells was also monitored at every time point by detecting p24 positive cells by flow cytometry. Interestingly, we found a significant reduction in the percentage of infected cells in selenium supplemented conditions in comparison to cells grown in Ctrl conditions (Figure 7e), at least at days 4 and 7 post-infection. Taken together, our data indicate no difference in HIV-1 replication in Jurkat by the supplementation of culture media with 100 nM selenium, except for a lower percentage of infected cells.

### 2.4. Selenium Levels Did Not Affect HIV-1 Replication in Primary Cells but Modified the Proportion of Infected Cells

**Figure 8:**
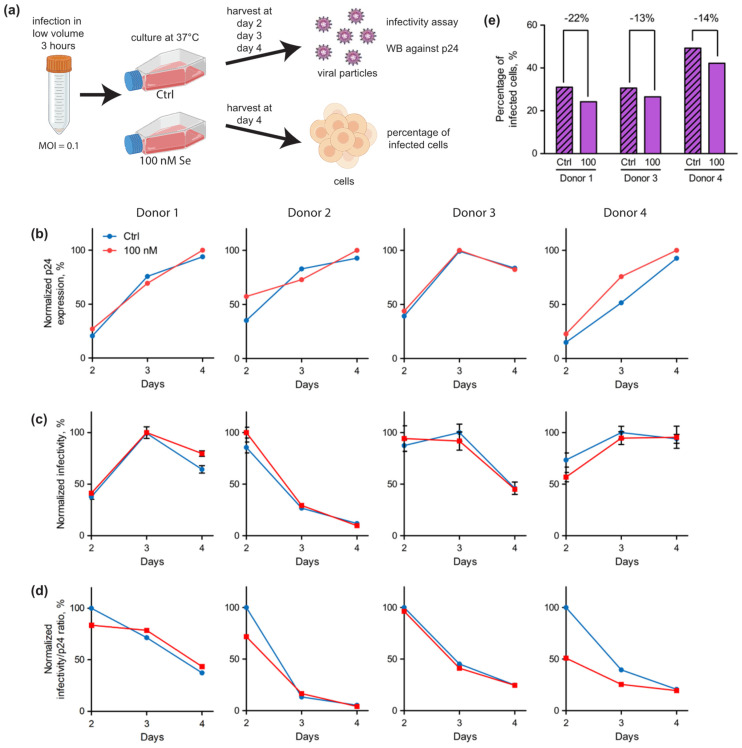
Kinetics of HIV-1 infection of T-cells isolated from donors in Ctrl or 100 nM supplemented conditions (**a**–**e**). (**a**) Schematic of the experimental procedure used to follow the different parameters of viral production and cell infection over four days. Similar to what was done with Jurkat in Figure 7, the media were collected at different time points and evaluated for the levels of p24 (**b**), infectivity of TZM-bl cells (**c**) and the ratio of infectivity over p24 levels (**d**) (*n* = 1). The infectivity assays were assessed with technical triplicates. For panels (**b**–**d**), the maximum value was arbitrarily set as 100%.

Since the selenoproteome of primary CD4 T-cells isolated from healthy donors seemed well responsive to selenium level variations and different from Jurkat cells’ selenoproteome, we also investigated whether selenium modified HIV-1 infection in this model. As these primary cells were less prone to infection than Jurkat cells, we used a higher MOI (0.1) than with the cell lines. Isolated CD4 T-cells were more susceptible to cell death after HIV-1 infection than Jurkat cells. Therefore, we performed shorter kinetics than with lymphoma-derived T cell infection (Figure 8a). Even then, at day 4 post-infection, for donor 2, cells could not be collected due to the very high rate of mortality. At days 2, 3 and 4 post-infection, aliquots of the culture media were harvested and evaluated for p24 production, and infectivity was assessed by a TZM-bl assay as described in the previous paragraph. The percentage of infected cells was measured when cells were harvested at day 4. As illustrated in Figure 8b, the production of viral particles detected by p24 Western blots were comparable with, or without, addition of selenium in the culture media, and this was observed for the four donors. Then, the infectivity of the collected particles from all donors was assessed on the TZM-bl cell line (Figure 8c). When comparing the particles produced in the presence or absence, of selenium supplementation, no significant difference could be detected for any of the donors. However, we could observe a small, but significant, effect in the proportion of infected cells when they were supplemented with selenium (Figure 8e). Taken together, our data indicate that the amount of virus particles were equally produced and infectious under the different experimental conditions in primary cells, but the percentage of infected cells was reproducibly lower in selenium supplemented conditions four days post infection. These data with primary cells are consistent with what we obtained with Jurkat cells.

### 2.5. The Infection of Primary T Cells with HIV-1 Altered the Levels of Certain Selenoproteins

**Figure 9:**
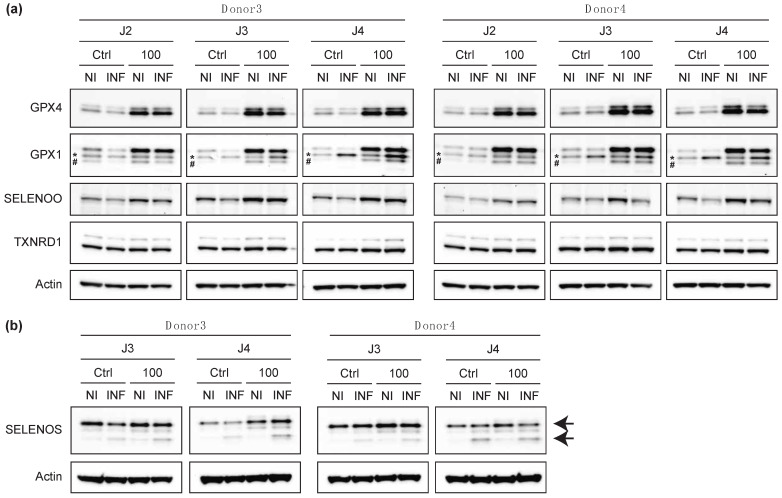
Analysis of selenoprotein expression in response to HIV-1 infection, in control (Ctrl) and 100 nM supplemented conditions. NI, non-infected cell extracts; INF, infected cell extracts. Donor 3 and Donor 4 were analyzed (*n* = 1) for the expression of SELENOO, GPX1, GPX4 and TXNRD1 (**a**) and SELENOS (**b**). Non-specific bands are indicated by an asterisk. The hash indicates an unknown band for GPX1. The arrows indicate the migration of two isoforms for SELENOS. The values are given in Table S5.

The fact that the selenium supplementation did not interfere with HIV-1 replication did not prevent the virus from altering the cellular use of selenium in selenoproteins. In this context, we therefore investigated whether HIV-1 infection affected the levels of selected selenoproteins in Ctrl and supplemented conditions (Figure 9). As we were limited in the biological samples available for this experiment, we performed this Western blot analysis with extracts from donors 3 and 4, for which we had a range of p24 positive cells between 30% and 50% (Figure 8e). With this level of infected cells, it would be difficult to visualize subtle effects. For example, in case of an inhibitory effect, the reduction in selenoproteome expression induced by HIV-1 infection would be diluted by the non-infected cells. On the other hand, an increase in expression or the emergence of a band corresponding to a protein isoform would be easier to observe. As illustrated in Figure 9, we monitored the expression of five selenoproteins, namely GPX4, GPX1, SELENOO, TXNRD1 and SELENOS, in response to HIV-1 infection at various time points and in cellular protein extracts. As a general trend, we observed a slight decrease in the expression of GPX1, GPX4, SELENOO and SELENOS at days 2, 3 or 4 post-infection. Interestingly, for SELENOS, an additional shorter form of the protein is visible at every time point post-infection and for the two donors tested here.

## 3. Discussion

Viruses use their host’s cellular machinery and metabolism to replicate and form new infectious particles. In turn, host cells have developed antiviral mechanisms to counteract or restrict viral replication. These anti-viral proteins are often referred to as restriction factors [54]. These proteins, which are considered the first line of defense against viral pathogens, are either upregulated by interferons (IFNs) or constitutively expressed. Interestingly, several of them have additional biological functions outside of immunity and can be envisioned as moonlighting proteins. The identification of cellular proteins able to restrict HIV-1 replication have received enormous research interest over the years, and several antiviral factors have now been clearly defined and include apolipoprotein B mRNA editing enzyme, catalytic polypeptide-like 3G (APOBEC3G), Tetherin, interferon-induced transmembrane proteins (IFITMs), cholesterol-25-hydroxylase (CH25H), KRAB-associated protein 1/ tripartite motif-containing protein 28 (KAP1/TRIM28), 90K, Moloney leukemia virus 10 protein (MOV10), myxovirus resistance gene B (MxB), Schlafen family member 11 (SLFN11), and zinc-finger antiviral protein (ZAP) [55]. However, viruses constantly evolve intricate solutions to evade or directly counteract many restriction factors, leading to continuous virus–host adaptation. The fact that selenium levels were associated with a decrease in CD4 T-cell counts in infected patients prompted us to hypothesize that selenoproteins could, in some way, also be involved in the control of HIV replication. As selenoproteins are principally regulated by selenium levels, we studied the impact of selenium addition on HIV-1 replication in different in vitro models available in the laboratory. Therefore, we started our work with two established cell lines (Jurkat and SupT1). Our data show that infection of a defined number of cells led to the production of similarly infectious viral particles, independent of the selenium concentration added. We then decided to confirm these results in primary CD4 T-cells that were isolated and activated from four healthy donors. Although primary CD4 T cells represent a more relevant model for HIV-1 infection, these cells are very difficult to grow and infect, and they are rapidly killed by the virus, rendering any biochemical analyses technically challenging. Data presented in Figure 8 confirmed that selenium had no significant effect on HIV-1 protein production nor infectivity. However, we observed that the concentration of selenium in the culture medium had a small, but significant, impact on the percentage of the cell population that is infected by the virus. Interestingly, we also confirmed in primary cells that HIV-1 infection altered the expression of cellular selenoproteins, as suggested in a pioneer experiment done in Jurkat with ^75^Se radioactive labelling with a decreased expression of several selenoproteins, including Gpx [46]. Our data suggest a link between selenium, selenoproteins and viral infection that we will investigate in future experiments.

The rationale of this work was to investigate the T-cell selenoproteome which is poorly described in the literature. Here, we first showed that expression of the selenoprotein mRNAs varied slightly between primary and established T cells, with high expression of GPX4, TXNRD1, SELENOT, GPX1, SELENOO, SELENOK, and SELENOW. As predicted from experiments that we performed in non-immune cellular models, synthesis of some selenoproteins was particularly sensitive to selenium variations in the culture medium at sub-micromolar ranges. This was particularly true for GPX1, GPX4, SELENOH, SELENOK and SELENOS, which are often referred to as stress-response members. On the other side, the selenoproteins that are part of the housekeeping members were less sensitive to changes in selenium concentration, and these include TXNRD1, SELENOP and SEPHS2. These data were then strengthened by measurement of enzymatic activities for GPXs and TXNRDs, which were enhanced by selenium supplementation, confirming that they represent an important line of the antioxidant defense and redox homeostasis in these immune cells. From our experiments, it appeared that SupT1 was less responsive to selenium variation than other cells, and this is why we selected Jurkat and primary cells to investigate the link between selenium and HIV infection in the following experiments. We were surprised to observe no significant effect of selenium on viral replication even when using primary cells, which is the relevant physiological model for HIV infection. It is noteworthy that we applied many experimental designs changing MOI, kinetics, and readouts but the conclusions remained unchanged. Surprisingly, it was previously shown that the overexpression of Gpx1 in SupT1 clones could accelerate HIV-1 replication, using very low MOI and rather long kinetics (14 days) [56]. In patients, infection with HIV-1 is a long and complex process that can be subdivided into three major phases: primary infection, which lasts for the first few weeks and is followed by a long latency phase (10–12 years) before the eventual progression to AIDS. As it was, our current experimental design mimics the first phase of infection (primary infection) with a high concentration of CD4 T cells that are infected in a short period of time and the virus that replicates rapidly. This contrasts with previous epidemiological studies, which investigated the fate of patients and the role of selenium during the latency. Thus, we do not see our results as being contradictory to previously published work. In fact, during HIV-1 latency, selenium most probably plays a role at many levels of the immune system or on many other cell types than CD4 T lymphocytes, such as macrophages, dendritic cells or reservoir cells, which have yet to be unambiguously identified. To our knowledge, this is the first demonstration that selenium concentration does not interfere with HIV-1 replication during the acute phase of replication but does not exclude a role in the later stages of the disease; this deserves further investigation.

Another interesting part of our finding concerns the changes in selenoprotein expression following HIV-1 infection in primary cells. Although we were faced with the technical challenge of working with non-transformed cells for which the culture is delicate and that are very inefficiently infected by the virus, we were still able to detect a slightly lower expression level for SELENOO, SELENOS, GPX4 and GPX1, whereas it was not the case for TXNRD1. In order to confirm these subtle changes at the selenoproteome scale, we compared our results with data obtained from mass spectrometry proteomics studies. Although these proteomics approaches continue to improve, only a few analyses have detected changes of T cells for low or moderately expressed cellular proteins in response to HIV-1 infection using mass spectrometry. Notably, a recent work described an elegant strategy to (i) circumvent confounding effects due to uninfected cells, (ii) limit the viral replication cycle to a single round and (iii) use a low MOI (i.e., ≤1) [57]. In this work, the authors infected primary human CD4 T lymphocytes with an HIV reporter virus encoding a cell surface streptavidin-binding affinity tag in place of the envelope (Env) gene, allowing antibody-free magnetic cell sorting of infected cells (AFMACS). This strategy allowed a rapid purification of HIV-infected cells from cultures, avoiding a tedious and potentially stressful cell sorting by flow cytometry. In this work, the authors were able to quantitate approximately 9000 proteins across multiple donors, among which they found 650 HIV-dependent changes. In this high coverage proteomic atlas, available as online supplementary data, we were able to find quantitative data for 15 members of the selenoproteome. In the three different donors, GPX1, GPX4, SELENOF, SELENOH, SELENOK, SELENOM, SELENON, SELENOO, SELENOS, SELENOT, SELENOW, SEPHS2, TXNRD1, TXNRD2, and TXNRD3 were detected, which is remarkable for a mass spectrometry proteomic approach and close to what could be obtained by immunological strategies. Among these selenoproteins, only TXNRD1 changed significantly in infected cell extracts in comparison to control conditions. At 24 and 48 h post-infection, a 15% and 25% decrease in protein expression was observed in infected cells, respectively. This decrease was in agreement with results obtained two decades ago using ^75^Se radioactive labeling [46]. This change was too subtle to be detected in our experimental design, using Western blots with less than 50% infected cells. However, the advantage of Western blot versus proteomics is the possibility of visualizing the emergence of protein isoforms, such as the one we observed for SELENOS after HIV infection. In their proteomic study, the authors also reported the downregulation of both GPX1 and GPX4 at 24 and 48 h, although this was not considered significant. The authors also studied proteomic changes observed in response to resting cell activation by TCR stimulation [57] and found a dramatic remodeling of the selenoproteome for the different donors with a significant decrease in SELENOW, SELENOH, SELENOM, GPX4, GPX1, SEPHS2, TXNRD2, SELENOF, TXNRD3 and SELENOO, and a significant increase in SELENOS, SELENON and SELENOK in response to T-cell activation. Only SELENOT and TXNRD1 remained unchanged. These data suggest a significant and differential function for several selenoproteins between resting and activated cells. However, it should be noted that detailed information about selenium concentration in the culture media used is missing, which restricts interpretation of the data. It would be interesting to recapitulate these experiments with a tight control of selenium concentration in order to understand its impact both on T-cell activation and selenoproteome remodeling during this process.

Another study reported the effects of HIV-1 infection on transformed T-cell lines (CEM), which were infected at high MOI with an Env-deficient, VSVg-pseudotyped virus, enabling a synchronous single round infection with less than 10% of uninfected cells [58]. In this work, only eight selenoproteins were detected that included GPX1, GPX4, SELENOF, SELENOH, SELENOS, SEPHS2, TXNRD1, and TXNRD2, but only very little changes were found. Only for SELENOH and SELENOS, a significant decrease (between 20% and 30%) was observed in the late phase of infection (48 and 72 h). Only in early time points (6 h) was a very weak decrease (between 12% to 15%) observed for GPX1, GPX4 and SEPHS2. Thus, this report and our data confirm that HIV-1 replication does not disrupt the selenoproteome during the acute phase of infection, but only induces subtle changes. Altogether, our results show that selenium concentration affects the expression of selenoproteins in T cells in a hierarchical manner, as it was described in non-immune cells. Amongst the proteins that are mostly affected, one can find GPX1, GPX4, SELENOH, SELENOK, SELENOS and SELENOT. However, in the context of our experimental setting that mimics only the early phases of HIV-1 infection, only very little effects or interplay with the selenoproteome could be observed.

## 4. Materials and Methods

This manuscript adopts the systematic nomenclature of selenoprotein names [59].

### 4.1. Materials

Jurkat, SupT1 and HEK293T cells lines used in this study were obtained from ATCC. TZM-bl cells were obtained from NIH AIDS Reagent program (Manassas, VA, USA). Fresh blood samples were provided by Etablissement francais du sang (EFS) de Lyon. Cell culture media and supplements, NuPAGE 4–12% bis–Tris polyacrylamide gels, MOPS and MES SDS running buffer, Dynabeads Human T-Activator CD3/CD28 were purchased from Life Technologies (ThermoFisher Scientific, Waltham, MA, USA). Fetal calf serum (FCS), sodium selenite, synthetic oligonucleotides, Percoll, Ficoll, t-BHP, NADPH, thioredoxin, L-GSH, glutathione reductase, DTNB, sucrose, DMSO, EDTA, Triton X100, glycerol and DTT were purchased from Merck (Darmstadt, Germany). Interleukin2 was from Eurobio Scientific (Les Ulis, France). The luciferase assay reagent was purchased from Promega (Charbonnières, France). The microplate readers FLUOSTAR OPTIMA and LUMISTAR OPTIMA were from BMG Labtech (Champigny-sur-Marne, France). The list of antibodies used in this study is given in Table S3. The plasmid pNL4.3 was obtained from the NIH AIDS Reagent program.

### 4.2. Cell Culture

Adherent cells (HEK293T and TZM-bl) were grown and maintained in 75 cm^2^ plates in Dulbecco’s Modified Eagle Medium (D-MEM). Cells in suspension (Jurkat, SupT1 and primary T cells) were cultured in Roswell Park Memorial Institute medium (RPMI). Media were supplemented with 10% fetal calf serum, 100 μg/mL streptomycin, 100 UI/mL penicillin, and 2 mM L-glutamine. Only for RPMI medium, 1 mM pyruvate and 10 mM HEPES were added. For the culture of primary T-lymphocytes, 30 U/mL Interleukin2 was added to the growth media. Cells were cultivated at 37 °C in humidified atmosphere containing 5% of CO_2_. Selenium supplementation was obtained by adding a defined volume of a concentrated solution of sodium selenite (0.1 mM diluted in ultrapure water) in the culture media. In our cell culture experiments, selenium is exclusively provided by the FCS, and can vary widely from providers and even from one lot to another of the same provider [49]. Here, we used a lot of FCS containing 110 nM of selenium, which is in the low range, and kept it for all the experiments of the present study. Therefore, the Ctrl growth conditions that contained 10% FCS (11 nM of selenium) could be considered selenium deficient.

### 4.3. Isolation and Culture of Primary CD4 T-Cells

We isolated human CD4 T cells from peripheral blood mononuclear cells (PBMC), using a negative selection strategy following the manufacturer’s instructions. Our studies were performed with fresh blood samples originating from different healthy donors. Briefly, peripheral blood mononuclear cells (PBMC) were isolated by Ficoll density gradient centrifugation, 25 min at 750× *g*, without the centrifuge brake. The PBMC ring was collected between the plasma and Ficoll and diluted with 1 × PBS. After several washes with 1 × PBS, the cells were counted, placed on a Percoll cushion and centrifuged for 20 min at 930× *g*. The peripheral blood lymphocytes (PBL) fractions were collected in the upper phase while the monocytes formed a ring at the interface. The PBL were counted, aliquoted at approximately 30 × 10^6^ cells/mL and stored at −80 °C in freezing medium composed of 10% DMSO and 90% SVF. The negative isolation of CD4 positive T cells was performed with the EasySep^TM^ Human CD4 T-Cell Isolation Kit (StemCell Technologies, Saint Égrève, France) according to the manufacturer’s protocol. Then, the activation of primary T lymphocytes via the T-cell receptor (TCR) complex was performed with Dynabeads Human T-Activator CD3/CD28 for 72 h according to the manufacturer’s protocol. After this time period of activation and expansion, the cells were then readily used for HIV-1 infection and/or selenium supplementation of the culture media.

### 4.4. HIV-1 Production in HEK293T Cells and Titration

HIV-1 viral particles were produced by transient calcium phosphate transfection of HEK293T cells with pNL4.3 plasmid DNA. The day prior to transfection, HEK293T cells were seeded at 6.5 × 10^6^ cells per 10 cm plates. Ten micrograms of pNL4.3 plasmid were transfected per plate using calcium phosphate precipitation of DNA. The medium was replaced by a fresh one 6 h later. Two days post-transfection, the medium was collected, filtered (0.45 µm cutoff) and concentrated by ultracentrifugation at 4 °C (1 h 30 min, 110,000× *g*) through a 20% sucrose cushion prepared in TNE buffer (10 mM Tris, pH 8, 100 mM NaCl, 1 mM EDTA). The concentrated viral particles from one 10 cm plate were resuspended in 100 μL RPMI, aliquoted and stored at −80 °C for several months. The viral particles were titrated by serial dilution on Jurkat cells (LTR HIV-1 GFP, from NIH AIDS reagent program).

### 4.5. Infection of CD4 T-Cells with HIV-1

Jurkat and primary CD4 T cells were counted and resuspended at 4 × 10^6^ cells/mL. A volume of freshly thawed HIV-1 viral particles was added to the cells to obtain a defined MOI (multiplicity of infection), previously calculated in Jurkat cells stably expressing GFP gene in the control of the HIV-1 LTR promoter. Infection occurred in a small volume for 3 h at 37 °C. The cells were then washed with 1 × PBS and resuspended in a culture medium, supplemented or not with 100 nM sodium selenite, and incubated at 37 °C from 2 to 15 days as indicated in every experiment.

### 4.6. Quantification of Infectious HIV-1 Produced by CD4 T Cells

The viral particles produced by CD4 T cells were analyzed for their capacity to infect TZM-bl cells. Previously designated JC53-bl (clone 13), the TZM-bl are HeLa cells producing large amounts of CD4 and CCR5 and separate integrated copies of the luciferase and beta-galactosidase genes under control of the HIV-1 promoter. We modified this cell line by integrating copies of the mCherry gene, using a lentivirus. The red fluorescence was directly proportional to cell numbers and therefore used to normalize the luciferase relative to cell density.

The day prior to infection, TZM-bl cell were seeded in a 96 well plate at 1 × 10^4^ cells/well. The day of infection, the collected media from CD4 T cells were serial diluted (1/3) in triplicate and added to the TZM-bl cells. After 48 h of growth, the cells were harvested and evaluated for red fluorescence and luciferase activity using a multiplate reader. In each well, the luciferase activity (AU) was expressed relative to the fluorescence intensity (AU). The infectivity of the media was then expressed relative to the maximum value of the experiment set at 100.

### 4.7. Quantification of p24 Contained in Viral Particles

To evaluate the quantity of viral particles released by CD4 T cells, aliquots of the culture media were collected and concentrated by 30 min ultracentrifugation at g though a 20% sucrose cushion at 4 °C. After removal of the supernatants, the pellets were resuspended in 20 µL of lysis buffer (25 mM Tris-HCl, pH 7.8, 2 mM DTT, 2 mM EDTA, 1% Triton X-100 and 10% glycerol). Equal volumes (10 µL) were separated in 4–12% Bis-Tris NuPAGE Novex Midi Gels and transferred onto nitrocellulose membranes using iBlot DRy blotting System (Themo Fisher Scientific, Waltham, MA, USA). Membranes were probed with indicated p24 antibodies and HRP-conjugated anti-mouse secondary antibodies. The chemiluminescence signal was detected using ECL Clarirty Max detection kit (Biorad, Hercules, CA, USA) in the Chemidoc Imager (Biorad). Data quantifications were performed with ImageLab Software (Biorad, Version 6.0.1, Hercules, CA, USA).

### 4.8. Total RNA Extraction and Analysis by RT-qPCR

The quantification of selenoprotein mRNAs was performed as described in [31]. Briefly, total RNAs were purified with Tri Reagent^®^ (Molecular Research Center, Cincinnati, OH, USA) according to manufacturer’s instructions and resuspended in ultrapure milliQ water. RNAs were reverse transcribed using qScript cDNA Synthesis kit (Quanta Bio, Beverly, MA, USA) according to the manufacturer’s instructions. Real-time PCRs were performed in triplicate using FastStart Universal SYBR^®^ Green master (Roche Applied Science, Penzberg, Germany) on a StepOne Real-Time PCR System (Applied Biosystems, Foster City, CA, USA). Primers used are described in [31] and listed in Table S6. Serial dilutions of a cDNA mixture were used to create a standard curve and determine the efficiency of the amplification for each pair of primers. The quantification of selenoprotein mRNAs was performed, using a set of four reference genes.

### 4.9. Protein Extraction and Analysis by Western Blot

After a wash with 1X PBS, cellular protein extracts were harvested with lysis buffer (described earlier). Then, protein concentrations were measured using the DC^TM^ protein assay kit (Biorad) in microplate assays. Equal protein amounts (30 µg) were separated in 4–12% Bis-Tris NuPAGE Novex Midi Gels and transferred onto nitrocellulose membranes using the iBlot^®^ DRy blotting System. Membranes were probed with indicated primary antibodies and HRP-conjugated anti-rabbit or anti-mouse secondary antibodies. The chemiluminescence signal was detected using an ECL Select detection kit (GEHealthcare, Chicago, IL, USA) in the Chemidoc Imager (Biorad, Hercules, CA, USA). Data quantifications were performed as described earlier.

### 4.10. GPX and TXNRD Enzymatic Assays

GPX and TXNRD activities were measured in enzymatic coupled assay as described in [37,38,60] with 30 μg of protein extracts. GPX enzymatic activities (U/mg) were measured with t-BHP substrate and expressed as nmol of glutathione/min·mg. TXNRD enzymatic activities (U/mg) were expressed as nmol of NADPH/min·mg.

### 4.11. Flow Cytometry

The percentage of infected cell was monitored by flow cytometry. Briefly, cells were washed once with PBS and fixed with PBS containing 4% formaldehyde for 20 min at 4 °C. Cells were washed once with PBS and once with staining buffer (PBS containing 2% fetal calf serum and 2 mM EDTA). Then, cells were incubated with anti-HIV-1 p24 antibody coupled with FITC diluted 1/100 in staining buffer, for 1 h at 4 °C. Cells were washed twice with staining buffer and analyzed on a FACS Canto (7 colors) using FACS Diva software (BD Biosciences) and analyzed with FlowJo software (Treestar, Ashland, OR, USA).

### 4.12. Ethics Statement

Primary blood cells were obtained from the blood of healthy donors (EFS-Lyon) in the form of discarded “leukopacks” obtained anonymously so that gender, race, and age of donors were unknown to the investigator and inclusion of women or minorities cannot be determined. This research is exempt from approval, although written informed consent was obtained from blood donors to allow use of their cells for research purposes.

## 5. Conclusions

HIV-1 infects CD4 T lymphocytes, monocytes, macrophages and dendritic cells, but only a decline in CD4 T cells reflects the progression of HIV infection. The fact that selenium levels were associated with a decrease in CD4 T-cell counts in infected patients justified the study of the impact of selenium on HIV-1 replication in laboratory models. We primarily characterized the expression pattern of the selenoprotein genes in two established cell lines (Jurkat and SupT1) and primary CD4 T cells isolated from the blood of anonymous healthy donors. We found that the expression of the selenoproteome in human T cells is tightly regulated by the selenium level of the culture media, and this is in agreement with reports from non-immune cells. We showed that selenium had no significant effect on HIV-1 protein production nor infectivity, indicating that selenium may not be involved in the acute phase of replication but does not exclude a role in the later stages of the disease since the selenium levels slightly modified the proportion of infected cells. Finally, the expression of the selenoproteome is slightly modified by HIV-1 infection.

## Data Availability

Requests for further information about resources, reagents, and data availability should be directed to the corresponding author.

## Supplementary Materials

The following are available online at https://www.mdpi.com/article/10.3390/ijms23031394/s1.
